# Silencing of Paternally Expressed Gene 10 Inhibits Trophoblast Proliferation and Invasion

**DOI:** 10.1371/journal.pone.0144845

**Published:** 2015-12-17

**Authors:** Haiying Chen, Manni Sun, Jing Liu, Chunxiao Tong, Tao Meng

**Affiliations:** Department of Obstetrics, The First Affiliated Hospital of China Medical University, Shenyang, People’s Republic of China; Sun Yat-sen University Medical School, CHINA

## Abstract

*Paternally expressed gene 10 (PEG10)* is an imprinted and monoallelic expressed gene. Previous study using a knockout mouse model revealed a crucial role of PEG10 in placental development, yet the exact function of PEG10 during placentation remains to be elucidated. In this study, denuded chorionic villi were prepared from first trimester human placentas, and transduced with PEG10 small interference RNA (siRNA) or non-targeting control sequence by lentiviral infection. Immunohistochemical staining revealed that silencing of PEG10 in the chorionic villous explants resulted in reduced immune-reactivity to CK7, Ki67 and integrin α5, implying that silencing of PEG10 impaired the proliferation of villous trophoblasts and may interfere with the activity of extravillous trophoblasts. We further investigated the role of PEG10 in the proliferation, migration and invasion of JEG-3 trophoblast cell line and the primary chorionic villous cells. PEG10-silenced JEG-3 cells and primary chorionic villous cells displayed a reduced proliferation rate and impaired invasiveness *in vitro*. Silencing of PEG10 in trophoblast cells led to upregulated expression of tissue inhibitor of metalloproteinase-1 (TIMP-1) as well as downregulated expression of matrix metalloproteinase (MMP)-2 and MMP-9. Furthermore, knockdown of TIMP-1 reversed the suppressed invasiveness of PEG10 siRNA-transduced JEG-3 cells. In conclusion, our study demonstrates that PEG10 plays an important role in trophoblast proliferation and promotes trophoblast invasion through TIMP-1.

## Introduction

*Paternally expressed gene 10 (PEG10)* is an imprinted gene derived from a retrotransposon that was previously integrated into the mammalian genome [1]. *PEG10* is characterized as a paternally expressed and maternally silenced gene localized in an imprinted gene cluster on human chromosome 7 and on mouse chromosome 6 [1,2]. Functional study of PEG10 using a knockout mouse model has revealed its crucial role in placental development [3]. Moreover, clinical studies have reported aberrant PEG10 expression in the human placenta in multiple pregnancy complications such as spontaneous miscarriages and fetal death [4,5], intrauterine growth restriction [6] and preeclampsia [7]. Although PEG10 has been proposed to be a critical player during gestation, its role in normal placental development and in the pathogenesis of the pregnancy disorders remains elusive.

The placenta is composed of various types of trophoblast cells with specialized functions [8]. Villous trophoblast (VT) cells proliferate and cover the mesenchyme and fetal vessels that form the placental villous tree. Subsequent fusion of VT generates an overlying syncytium of villous syncytiotrophoblast, which provides a large surface area for maternal-fetal nutrient and gas exchange. Extravillous trophoblast (EVT) cells arise from VT cells in trophoblast columns at the tip of villi. These cells do not proliferate, but invade the uterine spiral arteries as individual cells, remodeling the placental vasculature in order to allow sufficient placental perfusion. Failure to transform uterine arteries as a result of poor EVT invasion may cause inadequate placental perfusion, which associates with clinical manifestations such as recurrent miscarriage, fetal growth restriction and preeclampsia [9].

The invasion of the extracellular matrix by EVTs is a complex process [10]. Matrix metalloproteinases (MMPs) are zinc-dependent endopeptidases capable of degrading a wide spectrum of components of the extracellular matrix and facilitating cell invasion [11]. MMPs, particularly MMP-2 and MMP-9 which cleave type IV collagen, the main component of basement membrane, have been described in early embryonic and placental development [12–14]. The proteolytic activities of MMPs are precisely regulated, at least in part, by tissue inhibitors of metalloproteinases (TIMPs) [15]. TIMP-1 is a secreted glycoprotein that binds to the active site of MMPs on a 1:1 stoichiometic basis with preferential binding to MMP-9 [16]. The balance between MMPs and TIMPs locally at the invasive site is highly regulated in a spatiotemporal manner to guard EVT invasion during pregnancy.

In the present study, the function of PEG10 in trophoblasts was investigated in chorionic villous explants using a small interference RNA (siRNA)-mediated knockdown approach. The molecular mechanism underlying the regulatory role of PEG10 in trophoblast invasion was further characterized in JEG-3 trophoblast cell line and primary chorionic villous cells.

## Materials and Methods

### Preparation of human primary first-trimester chorionic villous cells and cell culture

Collection and experimentation of human tissues in this study were conducted in strict accordance with the protocols approved by the Clinical Research Ethics Committee of China Medical University, and written informed consent was obtained from all participants. Human placentas (gestation age of around 7 weeks) from women undergoing legal termination of normal pregnancy were collected at The First Affiliated Hospital of China Medical University. The areas of chorionic villi were isolated from the placentas, and the primary culture of chorionic villous cells was prepared based on a previously described method [17]. Primary chorionic villous cells were cultured in DMEM (Gibco, Carlsbad, CA, USA) supplemented with 10% fetal bovine serum (FBS, Hyclone, Logan, UT, USA), 200 U/ml penicillin and 200 μg/ml streptomycin, and maintained at 37°C in a humidified atmosphere consisting of 5% CO_2_.

JEG-3 trophoblast cell line was purchased from the Cell Bank of Chinese Academy of Sciences (Shanghai, China). JEG-3 cells were cultured in DMEM supplemented with 10% FBS at 37°C in an atmosphere of 5% CO_2_ for no more than 20 successive passages.

### Chorionic villous explant culture

Individual clumps of chorionic villi were dissected from human first trimester placentas (gestation age of around 7 weeks) under a dissection microscope, and washed with sterile PBS. In order to assess trophoblast proliferation and differentiation by immunohistochemistry, the layer of syncytriotrophoblasts was removed by incubating the chorionic villi in 0.125% trypsin for 5 min at 37°C based on a previously described method [18]. The tissues were recovered in FBS-free DMEM at 37°C for 2 h, and then infected with lentiviruses harboring *PEG10* siRNA or the control sequences. Twenty-four h after lentiviral infection, the tissues were laid on phenol red-free Matrigel (BD Biosciences, San Jose, CA, USA) in a Transwell upper chamber and cultured in virus-free DMEM containing 10% FBS, 200 U/ml penicillin and 200 μg/ml streptomycin in a 5% CO_2_ incubator at 37°C. For the ELISA assay on TIMP-1 secretion by the explants, the dissected chorionic villous fragments did not undergo syncytial denudation, but were directly subjected to lentiviral infection and *in vitro* culture as described above.

### Constructs and lentiviral transduction

Lentiviruses harboring *PEG10* siRNA, *TIMP-1* siRNA or non-targeting control (NC) sequences were purchased from HANBIO (Shanghai, China). The sequences encoding the short-hairpin RNA which contained the target sequences on *PEG10* mRNA or *TIMP-1* mRNA were inserted into the virus genome. The target site on *PEG10* mRNA was 5’-GCAGUCGGAGGAGAACAAC-3’, and the NC sequence was 5’-ttctccgaacgtgtcacgt-3’. The *TIMP-1* siRNA that was selected for the transduction and Transwell assay targeted on *TIMP-1* mRNA at 5’-gtcaaccagaccaccttat-3’.

JEG-3 cells, primary chorionic villous cells and denuded chorionic villi were infected with the indicated lentivirus(es) at a virus titer of 10:1 to the cell numbers. 8 μg/ml polybrene (Sigma-Aldrich, St. Louis, MO, USA) was added to the infecting medium to enhance infection efficiency. The cells or explants were cultured with the viruses for 24 h before replacing the medium with fresh culture medium.

### Real-time polymerase-chain reaction (PCR)

Total RNA was extracted with the RNAsimple^™^ Total RNA Extraction Kit (TIANGEN Biotech, Beijing, China) according to the manufacturer’s instructions, followed by reverse transcription to cDNA. Quantitative real-time PCR was performed using SYBR GREEN PCR Master Mix (Solarbio, Beijing, China) in an Exicycler^™^ 96 Real-Time Quantitative Fluorescence Analyzer (Bioneer, Daejeon, Korea). The real-time PCR primers are listed in Table 1. The expression level of each mRNA is expressed as the ratio to the level of *β-actin (ACTB)* mRNA, which is the internal reference in all real-time PCR tests.

**Table 1:** Real-time PCR primer sequences.

Name	Forward	Reverse
*PEG10*	CTCCAGCGTTGTTAGATGTT	GGACTGAAGATGAAATGCC
*MMP-2*	GGAATGAATACTGGATCTACTC	CTTCTTCACCTCATTGTATCTC
*MMP-9*	GCTACGTGACCTATGACATCCT	TCCTCCAGAACAGAATACCAGT
*TIMP-1*	TACACTGTTGGCTGTGAGGAATG	AGGCAAGGTGACGGGACTGG
*ACTB*	CTTAGTTGCGTTACACCCTTTCTTG	CTGTCACCTTCACCGTTCCAGTTT

### Western blotting

At 48 h post-infection, cells were lysed with NP-40 lysis buffer (Beyotime) to extract total proteins. The proteins were separated by SDS-PAGE and transferred to a PVDF membrane (Millipore, Bedford, MA, USA) which was then incubated with a specific primary antibody against human PEG10, MMP-2, MMP-9 or TIMP-1 (1:1000; Santa Cruz) at 4°C overnight. Following incubation with HRP-conjugated goat anti-rabbit IgG secondary antibody (1:5000; Beyotime), the target protein bands were visualized with the ECL solution (7SeaPharmTech, Shanghai, China). To verify equal loading and transfer, the membrane was stripped with the stripping buffer (Beyotime) and re-probed with anti-β-actin antibody (Santa Cruz).

### Immunohistochemistry (IHC)

The expression of the trophoblast marker, cytokeratin 7 (CK7) [18], the proliferative maker, Ki67, and the EVT makers, human leukocyte antigen (HLA)-G [19] and integrin α5 [18] in the chorionic villous explants was examined by IHC at 72 h after lentiviral transduction. The explants were fixed, paraffin embedded and sectioned. Following antigen retrieval, the sections were incubated sequentially with 3% H_2_O_2_, goat serum, and a specific primary antibody overnight at 4°C including anti-CK7 (1:100; Bioss, Beijing, China), anti-Ki67 (1:100; Bioss), anti-HLA-G (1:50; Santa Cruz) and anti-integrin α5 (1:200; Boster, Wuhan, China). Thereafter, the sections were incubated with biotin-conjugated goat anti-mouse/rabbit IgG and HRP-labeled streptavidin (Beyotime). Diaminobezidine (Sigma-Aldrich) was added for the chromogenic reaction, and the cell nuclei were stained with hematoxylin. The sections were mounted and observed under an Olympus DP73 microscope at 400× magnification.

### Cell proliferation/MTT assay

Cells were seeded in 96-well microplates at a density of 2×10^3^ cells/well at 24 h after lentiviral infection. MTT (Sigma-Aldrich) was added to the medium to a final concentration of 0.2 mg/ml at 24 h, 48 h and 72 h post-seeding, followed by 4 h incubation at 37°C. Thereafter, the supernatant was carefully aspirated, and 200 μl DMSO (Sigma-Aldrich) was added to each well to dissolve MTT-formazan crystals. Optical density (OD) values at 490 nm were measured in an ELX-800 microplate reader (BioTek, Winooski, VT, USA).

### Scratch assay

*In vitro* scratch wound assay, a well-established method to assess cell migration, was performed at 24 h after lentiviral infection as previously described [20]. The cells were observed and photographed under an inverted microscope at 0 h and 24 h post-scratching, and the percentage of wound closure, which reflects the migrating rate, was calculated as (original gap distance—gap distance at 24 h)/original gap distance ×100%.

### Transwell assay

At 24 h after lentiviral infection, cells were pre-treated with 5 μM mitomycin-C to inhibit proliferation. 1×10^4^ cells resuspended in 200 μl serum-free medium were plated in each Transwell chamber (Corning) pre-coated with phenol red-free Matrigel. The Transwell chambers were placed in 24-well plates containing 800 μl culture medium supplemented with 20% FBS in each well. After 24 h incubation in a 37°C incubator, the cells and the Matrigel on the top surface of the Transwell microporous membrane were wiped off with a cotton swab. The cells on the bottom surface of the membrane were fixed and stained with hematoxylin (Solarbio). Under a 200×inverted microscope, five fields on each membrane were selected, and the numbers of invading cells were counted.

### Gelatin zymography

The enzymatic activity of MMP-2 and MMP-9 was measured by gelatin zymography. Conditioned culture medium was harvested and centrifuged. The supernatant was mixed with 5× SDS loading buffer and subjected to SDS-PAGE containing 0.1% (w/v) gelatin in the gel. Thereafter, the gel was washed twice in 2.5% (w/v) Triton X-100 solution with shaking for a total of 80 min, and incubated in the incubation solution (50 mM Tris-HCl, pH 7.6, 5 mM CaCl_2_, 1 μM ZnCl_2_, 0.2 M NaCl) for 40 h at 37°C. The gel was then stained for 3 h with 0.05% Coomassie Blue R-250 (Amresco, Solon, OH, USA) in 30% methanol and 10% acetic acid, and distained in a series of graded methanol and acetic acid solutions until clear proteolytic bands appeared. The gel was imaged with WD-9413BN Gel Documentation & Analysis System (LIUYI, Beijing, China). Areas of proteolysis were visualized by the absence of staining in the gel and were quantified by densitometric analysis.

### Enzyme-linked immunosorbent assay (ELISA)

At 72 h after viral infection, the culture medium of chorionic villous explants was collected and centrifuged, and TIMP-1 levels in the supernatant was detected with an ELISA kit (USCN, Wuhan, China) according to the manufacturer’s instructions. The concentration of TIMP-1 in the medium was calculated from the OD values based on a standard curve.

### Statistical analysis

All experiments were performed with a minimum of three biological replicates, and data are presented as the mean ± standard deviation (SD). One way analysis of variance (ANOVA) followed by Bonferroni *post-hoc* test was used to analyze differences between multiple groups. GraphPad PRISM software (version 5.0; San Diego, CA, USA) was used for data analysis and plotting. The differences are considered statistically significant when *p*<0.05.

## Results

### Silencing of PEG10 suppressed VT proliferation and EVT differentiation in chorionic villous explants

**Fig 1:**
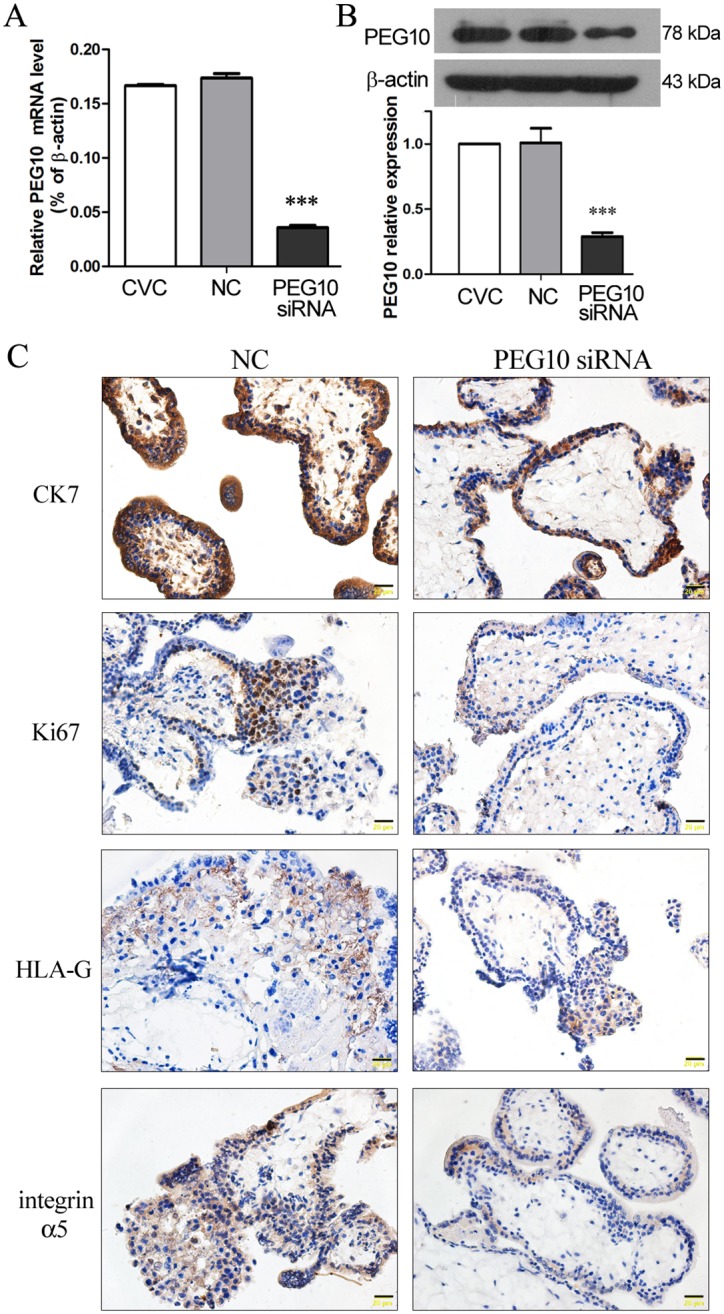
Silencing of PEG10 suppressed trophoblast proliferation and invasion in chorionic villous explants. (A, B) Primary human chorionic villous cells (CVC) were prepared from first trimester placentas and infected with lentiviruses harboring PEG10 siRNA or non-targeting control (NC) sequence. At 48 h after infection, the expression levels of (A) *PEG10* mRNA and (B) PEG10 protein were determined by real-time PCR and Western blot analysis, respectively. Data are expressed as the mean ± SD of three independent experiments. Significance versus non-transduced CVC, ***p<0.001. (C) Chorionic villi were prepared from first trimester human placentas, and subjected to syncytial denudation to remove the syncytiotrophoblast layer, followed by lentiviral transduction with PEG10 siRNA or NC. The expression of cytokeratin 7 (CK7), Ki67, HLA-G and integrin α5 in the chorionic villous explants was examined by Immunohistochemistry 72 h later. The sections were observed and photographed at 400× magnification (20 μm scales).

In order to investigate the role of PEG10 in trophoblast biology, *PEG10* siRNA was designed, and its knockdown efficiency was examined in the primary chorionic villous cells that were prepared from the chorionic villi of human placentas of around 7 gestational weeks. Lentivirus-mediated transduction of primary chorionic villous cells with PEG10 siRNA resulted in 75% reduction of *PEG10* mRNA and 71% reduction of PEG10 protein compared with the non-transfected cells, whereas transduction with NC sequences did not affect PEG10 expression (Fig 1A and 1B). These results indicated an efficient silencing of PEG10 expression in primary chorionic villous cells via lentiviral transduction of PEG10 siRNA.

Chorionic villi were dissected from human placentas of around 7 gestational weeks, and the layer of syncytiotrophoblasts was removed by trypsinization to allow trophoblast proliferation and differentiation [18]. Following syncytial denudation, the villi were infected with lentiviruses harboring *PEG10* siRNA or NC sequences, and the proliferation and differentiation of trophoblasts were assessed by IHC at 72 h after viral infection. As shown in Fig 1C, there were generally 3–4 layers of CK7-positive trophoblast cells at the edge of the NC-transduced villous explant, whereas there were only 1–2 layers of CK7-positive cells in PEG10-silenced villous explant, suggesting that trophoblast proliferation was inhibited upon PEG10 silencing. Moreover, trophoblast proliferation was also assessed by Ki-67, and the strong proliferative capacity of the cells at the edge and the proximal part of the control explant was not observed in PEG10-silenced explant. The immuno-reactivity to HLA-G, an identifying marker of EVT [19], was comparable between NC-transduced explant and PEG10-silenced explant. However, the immuno-reactivity to integrin α5, which is highly expressed in invasive EVTs [18], was reduced in PEG10-silenced explant compared with the NC explant. The IHC results suggest that PEG10 is required for VT proliferation, and it may also play a role in EVT differentiation.

### Silencing of PEG10 inhibited proliferation, migration and invasion of trophoblast cells

**Fig 2:**
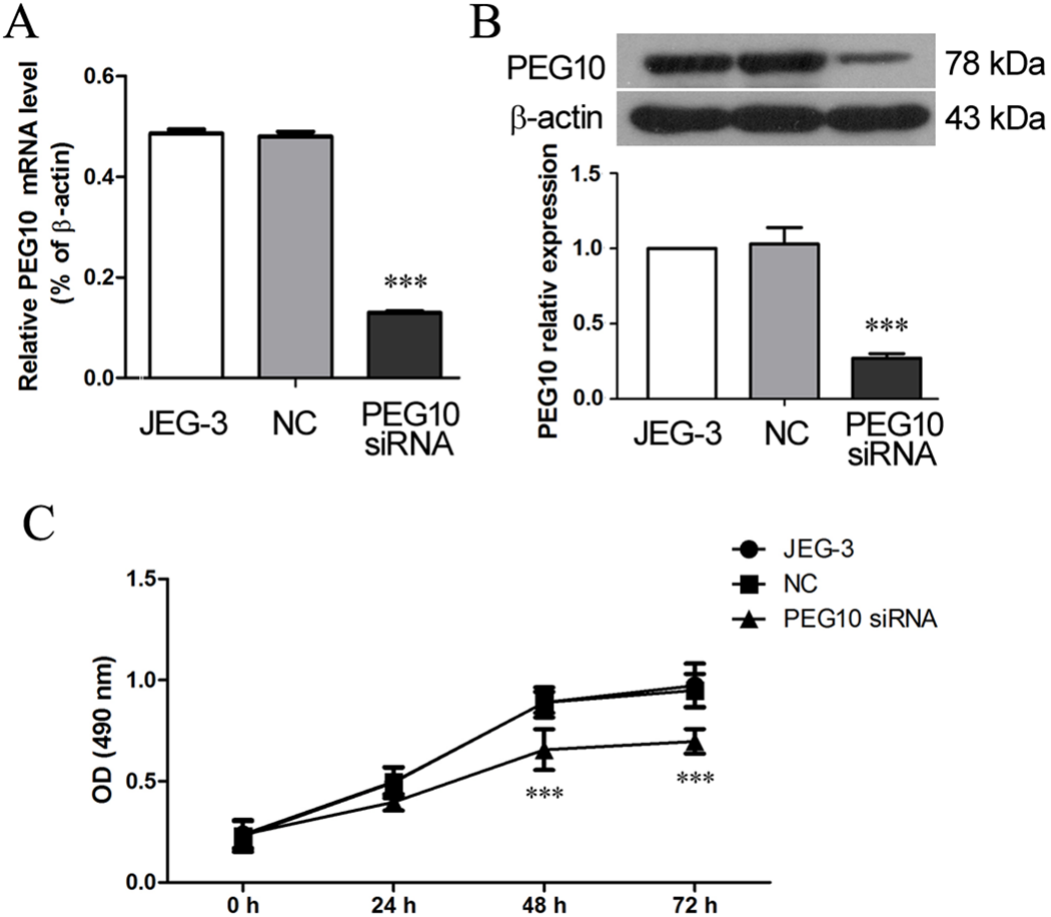
Silencing of PEG10 suppressed proliferation of JEG-3 cells. JEG-3 cells were infected with lentiviruses harboring PEG10 siRNA or NC sequences. At 48h post-infection, (A) *PEG10* mRNA level and (B) PEG10 protein level was determined by real-time PCR and Western blotting, respectively. (C) MTT assay was performed to assess cell proliferation with 5 replicates at each time point. Data are expressed as the mean ± SD of three independent experiments. Significance versus non-transduced JEG-3 cells, ***p< 0.001.

To study the molecular mechanism underlying the role of PEG10 in trophoblast behavior, JEG-3 trophoblast cell line was employed. JEG-3 cells were transduced with *PEG10* siRNA which achieved 76% reduction in *PEG10* mRNA level (*p*<0.001) and 71% reduction in PEG10 protein level (*p*<0.001) 48 h after lentiviral transduction, as compared with the non-transfected cells (Fig 2A and 2B). The MTT proliferation assay revealed that *PEG10* siRNA-transduced JEG-3 cells exhibited a decreased proliferative capacity, whereas the NC sequence did not affect JEG-3 proliferation (Fig 2C).

**Fig 3:**
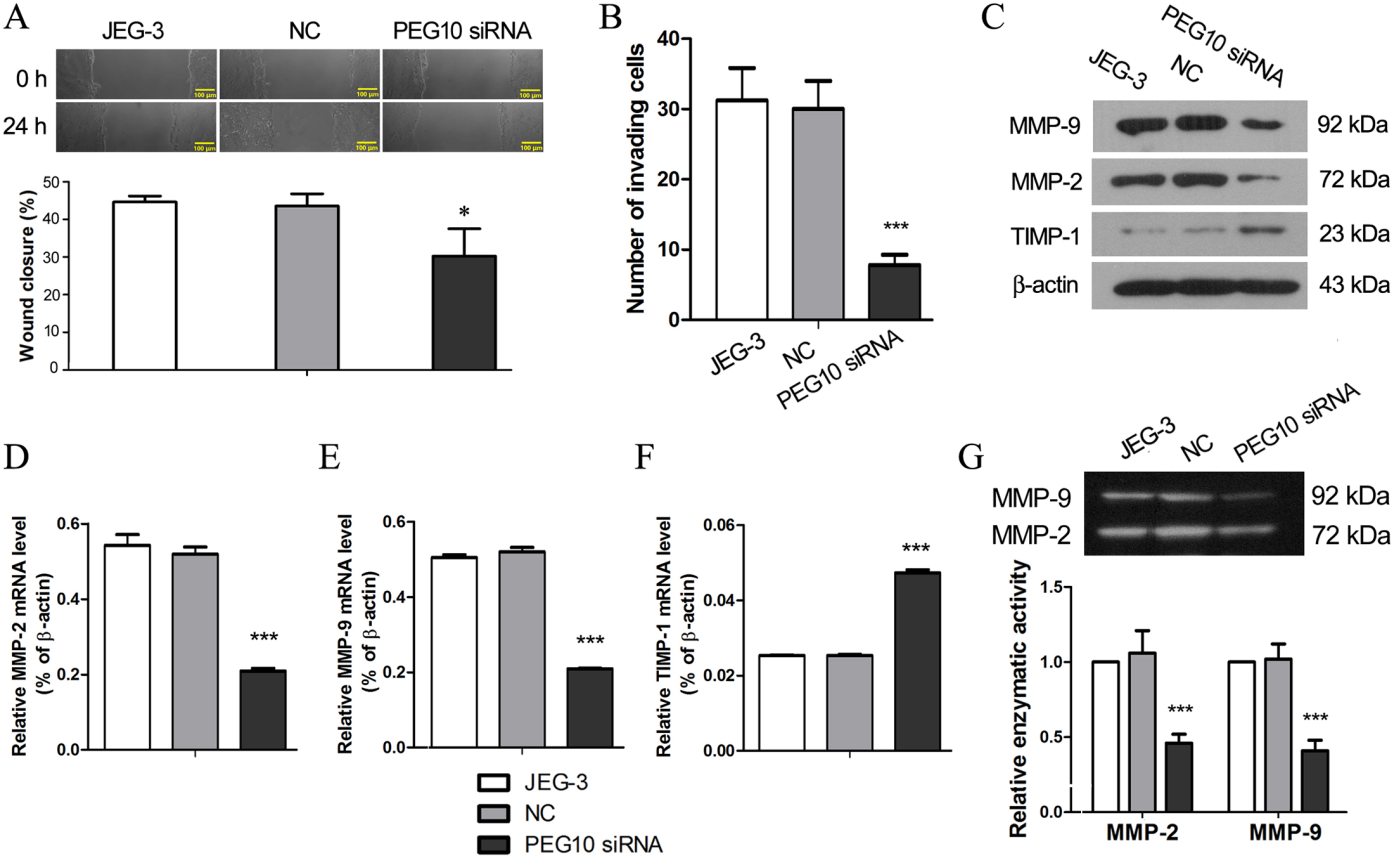
PEG10 silencing inhibited migration and invasion of JEG-3 cells. (A) Migration of PEG10-silenced JEG-3 cells was evaluated by *in vitro* scratch wound assay. (B) Matrigel Transwell assay was performed to assess invasiveness of PEG10-silenced JEG-3 cells. Expression of the key players of cell invasion including MMP-2, MMP-9 and TIMP-1 was examined by (C) Western blotting and (D-F) real-time PCR. (G) Gelatin zymography was performed to determine the enzymatic activities of MMP-2 and MMP-9 which were from the conditioned culture medium. The figure shows the representative images of three independent experiments and data are expressed as the mean ± SD. Significance versus non-transduced JEG-3 cells, *p< 0.05, ***p< 0.001.

The effects of *PEG10* silencing on trophoblast migration and invasion were assessed in JEG-3 cells by *in vitro* scratch wound assay and Matrigel Transwell assay, respectively. Silencing of PEG10 inhibited JEG-3 migration, as indicated by a lower wound closure rate compared with the control cells (*p*<0.05; Fig 3A). Moreover, the invasiveness of PEG10-silenced JEG-3 cells was remarkably reduced (*p*<0.001; Fig 3B), suggesting that PEG10 is required to promote trophoblast invasion. The key players of trophoblast invasion were further investigated. The expression of MMP-2 and MMP-9 were reduced approximately by half at both mRNA an protein levels in PEG10-silenced JEG-3 cells, while their inhibitor, TIMP-1, was upregulated by over 2 folds upon PEG10 silencing (*p*<0.001; Fig 3C–3F). In addition, using gelatin zymography, we found that the proteolytic activities of MMP-2 and MMP-9 were decreased in the conditioned medium of PEG10-silenced cells (*p*<0.001; Fig 3G), which may be largely attributed to the reduced protein secretion by these cells. Collectively, these results provided the molecular evidence for the compromised invasiveness of PEG10-silenced JEG-3 cells.

**Fig 4:**
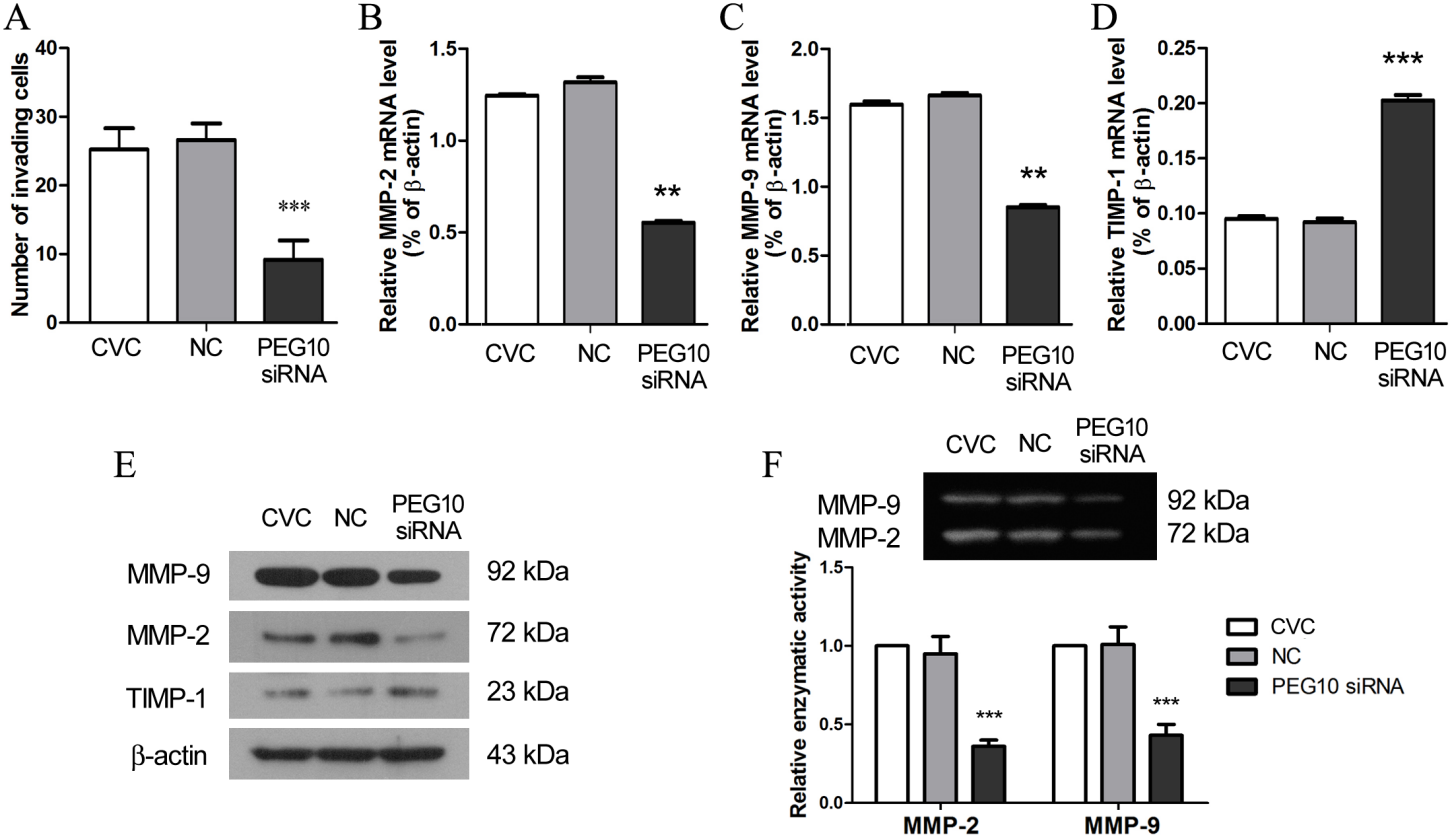
PEG10 silencing inhibited invasion of primary chorionic villous cells. (A) The invasiveness of PEG10-silenced primary chorionic villous cells (CVC) was assessed by Matrigel Transwell assay. (B-E) The expression of MMP-2, MMP-9 and TIMP-1 in PEG10-silenced primary CVC was examined by real-time PCR and Western blotting. (F) The enzymatic activities of MMP-2 and MMP-9 were measured by gelatin zymography. The figure shows the representative image of three independent experiments, and data are expressed as the mean ± SD. Significance versus non-transduced CVC, **p< 0.01, ***p< 0.001.

To verify the results obtained from JEG-3 cell line, the function of PEG10 in trophoblast migration and invasion was further examined in primary chorionic villous cells that mainly comprised of trophoblasts. Consistent with the observations in JEG-3 cells, PEG10 silencing profoundly suppressed the invasiveness of primary chorionic villous cells (Fig 4A). At molecular level, PEG10 silencing resulted in significant downregulation of MMP-2 and MMP-9 while upregulated the expression of TIMP-1 (Fig 4B–4E). Furthermore, the proteolytic activities of MMP-2 and MMP-9 were reduced in primary chorionic villous cells as a result of PEG10 silencing (Fig 4F). These results suggest that PEG10 plays a suppressive role in trophoblast invasion.

### Silencing of TIMP-1 reversed PEG10 siRNA-induced inhibition of trophoblast invasion

**Fig 5:**
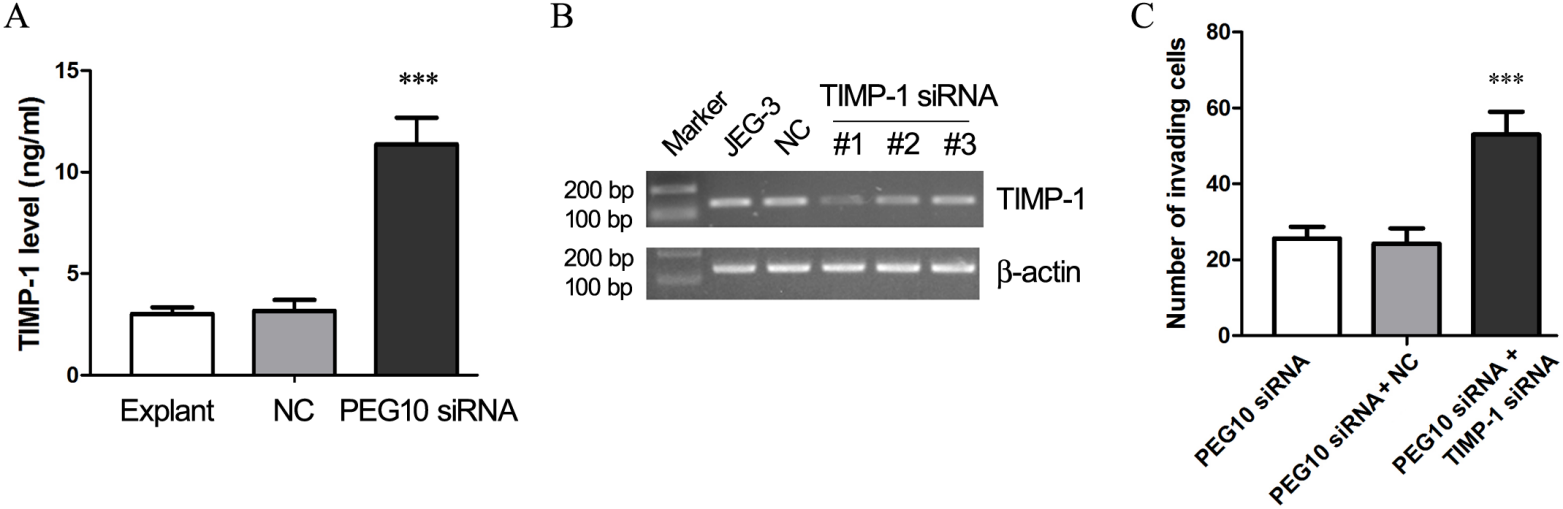
Silencing of PEG10 elevated secretion of TIMP-1 by chorionic villous explants and knockdown of TIMP-1 reversed PEG10 siRNA-induced inhibition of JEG-3 invasion. (A) The levels of TIMP-1 in the culture medium of chorionic villous explants were measured by ELISA at 72 h after lentiviral infection. Data are expressed as the mean ± SD of three independent experiments. Significance versus non-transduced explants, ***p< 0.001. (B) Three sets of *TIMP-1* siRNA were designed and transduced into JEG-3 cells, and 24 h later, the level of *TIMP-1* mRNA was assessed by PCR. (C) JEG-3 cells were transduced with *PEG10* siRNA alone, or double-transduced with *PEG10* siRNA and *TIMP-1* siRNA, and subjected to Transwell assay at 24 h after lentiviral infection. Data are expressed as the mean ± SD. Significance versus *PEG10* siRNA-transduced JEG-3 cells, ***p< 0.001.

We had observed upregulation of TIMP-1 in *PEG10* siRNA-transduced JEG-3 and primary chorionic villous cells, and we further assessed the secretion of TIMP-1 by chorionic villous explant. At 72 h after lentiviral infection, the level of TIMP-1 in the explant culture medium was examined by ELISA assay. The results indicated that *PEG10* siRNA-transduced chorionic villous explants secreted significantly more TIMP-1 into the medium than the control explants did (*p*<0.001; Fig 5A). The increased expression of TIMP-1 in the chorionic villous cells/explants lacking PEG10 led us to the hypothesis that reduction of TIMP-1 may rescue the defective invasiveness of PEG10-deficient trophoblast cells. Hence, 3 distinct TIMP-1 siRNA sequences were designed and transduced into JEG-3 cells via lentiviral infection, and the one with the highest potency to suppress TIMP-1 expression (Fig 5B) was used for the follow-up Transwell assay. The results showed that double transduction with *TIMP-1* siRNA and *PEG10* siRNA enhanced the invasiveness of JEG-3 cells that were transduced with *PEG10* siRNA alone (Fig 5C), implying that silencing of TIMP-1 could reverse the impaired invasiveness that resulted from reduced PEG10 expression.

## Discussion

Despite the increased interest on human imprinted genes and the recognition of the importance of the epigenetic apparatus in human development, the knowledge about the roles of the imprinted genes in human placental development and embryogenesis is limited. By analyzing the parent of origin SNPs of PEG10 in 10 family trios, Metsalu et al. identified that PEG10 was exclusively expressed from the paternal allele in the placenta, although the child’s blood exhibited a heterogeneous genotype in the same position, confirming the imprinting of the maternal allele of PEG10 in the human placenta [21]. Aberrant expression of PEG10 in the placenta was found clinically to be associated with several pregnancy complications [4–7], suggesting a potential role of PEG10 in placental development and function. In the present study, with a siRNA-mediated knockdown approach, we show that PEG10 affects trophoblast proliferation, differentiation and invasion, and that PEG10 regulates trophoblast invasion via MMP-2, MMP-9 and TIMP-1. Imprinting may serve as the epigenetic regulation of gene expression, yet the mechanism underlying the deregulation of placental PEG10 in pregnancy complications is to be investigated in future studies. Moreover, whether the expression of the maternal allele is activated in case of paternal allele expression failure is also an interesting aspect to look at.

PEG10 is predominantly expressed in the placenta of both human and mouse. Mouse embryo lacking PEG10 appeared normal and the placenta was slightly smaller than that of wild type at E9.5, but the placenta became severely depleted at E10.5 when the embryo displayed serious growth retardation and loss of heartbeat [3]. In humans, the expression of PEG10 is low at the early hypoxic phase but is highly activated at approximately 11–12 weeks of gestation [22], implying that PEG10 is required for placental development at a later stage of the first trimester. Dynamic expression of PEG10 at different stages of pregnancy was observed both in normal placental development and in spontaneous miscarriage and fetal death [4]. Hence, examination of PEG10 levels after delivery of placentas may not reveal the pathogenic mechanism for the pregnancy complications because of the lapsed action time. For example, contradictory PEG10 levels in preeclamptic placentas were reported [7,23], presumably owning to the placentas at different gestation ages. In the present study, primary chorionic villi were isolated from the human placentas at a gestation age of around 7 weeks, approximately when PEG10 starts to function. Therefore, our study is assumed to reveal the true functions of PEG10 in trophoblast behavior during early pregnancy. The major limitation of our study, on the other hand, is the impurity of the primary EVTs when investigating the molecular mechanism for PEG10-regulated EVT invasion, and this is mainly due to the limited amount of the materials. Follow-up studies on the role of PEG10 in each lineage of trophoblasts will be conducted in future with the specific lineages.

The factors that control VT proliferation and trophoblast differentiation into motile EVT are yet unclear. In the present study, silencing of PEG10 in the chorionic villous explant led to suppressed VT proliferation as revealed by IHC for CK7 and Ki67. On the other hand, the immune-reactivity to integrin α5 was reduced in PEG10-silenced villous explant compared with the control explant. These results suggest that PEG10 may play a critical role in VT proliferation, and it may be also involved in EVT differentiation. Further functional analysis revealed that silencing of PEG10 inhibited migration and invasion of JEG-3 and chorionic villous cells, suggesting that PEG10 may play an important role in EVT invasion, and that EVT dysfunction in the PEG10-deficient placenta may contribute to the pathology of some pregnancy complications.

MMPs were found highly expressed in EVTs which invade uterine arties to remodel placental vasculature [9]. PEG10 has been shown to promote the expression of MMP-2 and MMP-9 in cancer cells for an enhanced invasiveness [24,25]. Here we demonstrated that the invasiveness was inhibited in PEG10-silenced JEG-3 cells and primary chorionic villous cells, associating with downregulation of MMP-2, MMP-9 and upregulation of TIMP-1. These data suggest that PEG10 may promote EVT invasion by modulating the ratio of MMPs and TIMP-1. In addition, reduction of TIMP-1 expression by siRNA reversed the impaired invasiveness of the JEG-3 cells lacking PEG10, reinforcing the notion that TIMP-1 is a downstream target of PEG10 in the regulation of trophoblast invasion. EVT cells are often compared with highly invasive carcinoma cells due to their inherently invasive nature, thus it is not surprising that they share similar regulatory mechanisms for cell invasion. Wnt/β-catenin pathway was postulated be a downstream target of PEG10 in tumor metastasis [25]. However, the precise molecular mechanism for PEG10-regulated EVT invasion is to be elucidated in future studies.

In conclusion, our study demonstrates that silencing of PEG10 inhibited trophoblast proliferation and invasion, and led to upregulation of TIMP-1 and downregulation of MMP-2 and MMP-9. The data suggest that PEG10 may play a critical role in promoting EVT invasion during early pregnancy.
